# Effect of Salicylic Acid and Pre-Cold Treatment on Flower Induction in Saffron

**DOI:** 10.1155/2022/6108161

**Published:** 2022-10-21

**Authors:** Sakineh Rastegari, Seyed Mehdi Naser Alavi, Mehdi Mohayeji

**Affiliations:** Shahid Bahonar University, Kerman, Iran

## Abstract

Saffron is an important flowering plant, generally known as a golden condiment. The present study was performed to find the influence of different levels of SA and pre-cold treatment in the dormancy period of saffron and their effects on content enzyme activity. The results indicated that the SA2%, SA1%, and, pre-cold treatments took the shortest day to flowering. PAL enzyme activity was highest in pre-cold treatment. The higher total amount of protein was measured in the control, SA1% and SA2%. The highest amount of starch content and total soluble sugar was detected in pre-cold, SA2%, and control, respectively. No significant differences between treatments were present for CAT, PPO, GPX, and APX enzymes activity. There was a negative significant correlation between flowering time and some studied traits, i.e., starch and PAL activity. Applying SA and pre-cold treatment can induce saffron flowering and effect on pal enzyme activity and corm total protein, sugar, and starch content accordingly.

## 1. Introduction

Saffron (*Crocus sativus* L) or red gold is one of the most valuable and expensive plants in the world [1]. The dried stigma of this plant, which is known as saffron, has a diverse range of usage from food industries to traditional medicinal uses. Saffron is a sterile triploid (2*n* = 3*X* = 24) plant that its corm can endure a dry dormant period. Flowering stage in saffron occurs during autumn (October-November) then the vegetative stage begins during winter and pending this period, the formation of replacement corms would happened at the base of the shoots. In saffron, flower emergence occurs in the range of 10–14°C [2]. The leaves senesce and wither at the beginning of the dry season and then corms go into the dormancy [3]. Corm dormancy period includes two stages: real dormancy, in which activity of the plant is at the lowest level (early May to the middle of January). Initiation and differentiation of the flower buds and leaves occur in the second stage, which is known as pseudo dormancy (mid-January to mid-October) [4].

Some exogenous growth regulators affect on the various stages of plant growth, dormancy, and flower induction as well as endogenous hormones [5]. Plant growth regulators (PGRs) play a key role to control flowering time in the plants. Along with endogenous hormones, exogenous application of PGR(s) can interact between plant promoters and inhibitors substances and therefore regulate growth and the dormancy stages in plants [5]. There are different ways for PGR application such as soaking and foliar application. Soaking bulbs in the solution of chemical compound due to its dosage, time consumption, and cost is an efficient method for obtaining good results [6].

Salicylic acid (ortho-hydroxybenzoic acid) is one of the most notable important metabolites in plants which belongs to phenolic compounds [7]. It has a diverse range of performances in the world from curing various human illnesses to protecting plants from various biotic and abiotic stresses [8]. Salicylic acid is the phytohormone that plays important roles in germination, leaf senescence, fruit ripening, thermogenesis, flower induction, ethylene biosynthesis, stomatal respiration, behavior growth, and development in many plants [9].

Corm storage temperature is one of the factors influencing saffron production [3]. Some previous studies have reported that saffron production is affected by the characteristics of the growing area and the storage temperature of the corms [10]. Among all environmental factors, the role of temperature during the growth and development of saffron is very important [11].

By shortening the corm dormancy period, flowers appear before getting cold, and it helps to avoid frost damage [12]. The goal of this study was to investigate the effect of pre-cold treatment and different levels of SA on saffron flowering time and changes in total soluble sugar, starch content, and enzymatic activities in saffron corm.

## 2. Material and Method

An aeroponic experiment was carried out as a completely randomized design with three replications at the greenhouse of agriculture faculty, Shahid Bahonar University of Kerman, Iran. Saffron corms were prepared in the middle of July 2020 from Zarand, Kerman. After cleaning and weighting, corms with the average weight of 12–14 g with no contamination and mechanical damages were selected for further experiment. Then, they were divided into three groups, one group was soaked in a solution of SA (produced by Merck) 1% and 2% for 24 hours and the other was stored at 4°C for 28 days for pre-cold treatment and the third group was considered as control. The soaked corms were left to dry in room condition and then disinfected with Carbendazim, and this stage was repeated for control treatment and pre-cold treatment after 28 days. Then, saffron corms were transferred to the greenhouse. The environmental conditions in the aeroponic greenhouse were set up under dark condition with a relative humidity of 75%–80% and temperature range of 20–22°C. After six weeks, the temperature had been reduced to 15°C for sprout induction. During this growth stage, the daily light was changed to 18–6 light-dark.

### 2.1. Extract Preparation

Extracts were prepared from corms. Briefly, corms were homogenized in phosphate buffer 50 mM, pH 7.2, containing PVP 1%, and EDTA 1 mM. After centrifugation at 10000 × g for 30 min, a clear, transparent supernatant termed “crude extract” was obtained and used for further experiments.

### 2.2. Protein and Enzyme Assay

Total protein was measured by the method of Bradford [13] using a protein-dye reagent and bovine serum albumin as a standard. Catalase (CAT) activity was measured according to the method of Dhindsa et al. [14] with some modifications spectrophotometrically, by monitoring the decrease in absorbance at 240 nm resulting from the decomposition of H_2_O_2_. Polyphenol oxidase (PPO) activity assay was performed with 50 mM phosphate buffer (pH 7.2), 0.02 M pyrogallol, and 100 *μ*L of the crude extract by measuring the increase in absorbance at 420 nm for 4-methyl catechol spectrophotometrically, using Bio Tek Multivolume spectrophotometer [15]. For ascorbate peroxidase (APX), 50 mM phosphate buffer (pH 7), 0.5 mM ascorbate, 0.15 mM H_2_O_2_, 10 mM EDTA, and 150 *μ*L of crude extract were mixed. The rate of oxidation of ascorbate can be detected by the decrease in absorbance at 290 nm with spectrophotometer [16]. The activity of Guaiacol peroxidase (GPX) was measured spectrophotometrically as described by Plewa et al. [17]. The assay mixture contained 50 mM phosphate buffer, H_2_O_2_ 3%, and Guaiacol 99%. Due to the formation of tetra-guaiacol GPX activity was measured in absorbance at 470 nm. PAL enzyme activity was measured using a spectrophotometric assay. Enzyme extracts were prepared according to Hahlbrock and Schröder [18] method with some modifications. PAL activity was defined by the production of cinnamate and measured by absorbance change using a Bio Tek multivolume spectrophotometer. Specific enzyme activity was expressed as micromole of cinnamic acid produced per minute and per milligram of protein.

### 2.3. Total Sugar and Starch Content

Total soluble sugar and starch were assayed with the spectrophotometry method according to anthrone method [19] with some modifications. 0.5 gram of corm tissue was extracted in ethanol (80%). Then, samples were placed in a water bath 70°C for 10 minutes and were centrifuged for 15 minutes at 5000 rpm. In order to determine the total soluble sugar, 3 ml anthrone was added to the 100 *μ*l of alcohol extract. The extract was placed in the water bath for 10 min again to react. After cooling, absorption at 625 nm was read with Bio Tek multivolume spectrophotometer. For starch measurement, the residue of sugar tissue was washed by distilled water and then 10 ml H_2_SO_4_ 98% were added and were centrifuged for 15 minutes at 5000 rpm. Afterwards, anthrone regent was added. After extract reaction with anthrone reagent in water bath and getting cool, starch was read in 630 nm by using spectrophotometer.

### 2.4. Statistical Analysis

The data were statistically processed by analysis of variance (ANOVA) according to a completely randomized design, and means with standard errors were calculated using the program SPSS, version 26.0. Differences between the treatments were determined using Dunkan's multiple range test at the *P* < 0.05 probability level. Pearson correlation also was assessed for all measured traits by SPSS program as well.

## 3. Results and Discussion

### 3.1. Day to Flowering

**Figure 1:**
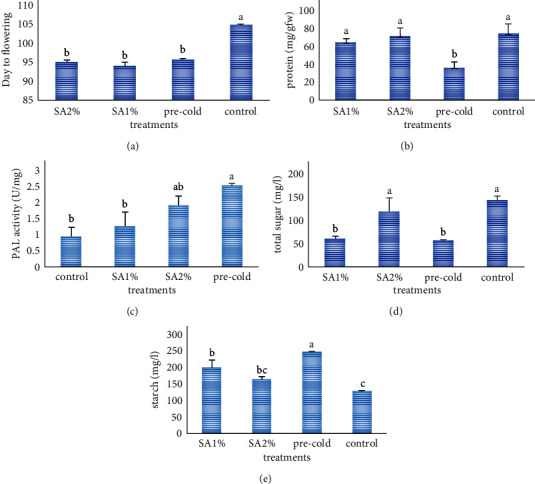
The effect of pre-cold and different levels of SA on (a) day to flowering; (b) corm total protein; (c) PAL activity; (d) com total soluble sugar content; (e) corm starch content.

The effect of SA and pre-cold treatments on the trait of day to flowering was highly significant (*P* < 0.001) (Table 1). Flowering occurred earlier in SA2%, SA1%, and pre-cold treatments, and control treatment had a few days delay in flowering (Figure 1(a)). The experiment showed that the application of SA as a PGR and pre-cold treatment in the act of a physical treatment could decrease the length of period of dormancy and stimulate flowering in comparison to control treatment. Flower-inducing and bud formation was the first indication of a physiological effect of SA in tobacco cell cultures which was reported by Eberhard et al. [20]. With regards to the demonstrated stimulatory effect of SA on flowering in different plant species, Popova et al. [8] suggested that SA can act as a plant flower regulator. In fact, SA as a PGR can form a major signaling network that transmits external or internal changes and contributes to the extraordinary ductility of the flowering process [21], such as phytohormone signaling pathways. Also, it regulates a few numbers of flower-integrating genes, such as *FT* and *SOC1*, which activate the flowers meristem identity genes [22]. SA is known as a flowering promoter in plants and can influence on plant flowering time markedly [23]. Hayat et al. [24] reported flower-inducing in *L. gibba* in the presence of SA analogs. The same as our result, SA could induce flowering when exogenously applied to the Lemnaceous plants [25]. According to their results, it suggested that SA is a plant flowering regulator and can accelerate vegetative to reproductive phases transition, which may be the reason of earlier flowering in our experiment. In the present study, the highest amont of day to flowering was belonged to control. SA is an important factor in flowering time as Hayat et al. declared that under no stress condition or exogenoused hormone, SA genes pathway express at low level.

Dole [26] expressed that there is no cold requirement for breaking dormancy in saffron corms, and Noy-Porat et al. [27] declared that in some bulbous flowers, all stages of florogenesis ceased in low temperatures, and *NtFT* expression is inhibited. However, our experiment showed that low temperature could reduce the length of dormancy period and stimulates flowering. Hajyzadeh et al. [28] reached the same result as ours and indicated that cold-stored corms had earlier flower induction in saffron. However, other data, which were not listed here, showed no preference to use pre-cold treatment.

### 3.2. Total Protein

Significant difference (*P* < 0.05) was determined in total protein (Table 1). SA2%, SA1%, and control treatments were classified in the same group with the highest amount of protein, while pre-cold had the lowest amount of protein (Figure 1(b)). Janda et al. [29] referred that photosynthesis, lignin, leaves pigments, and protein content can be affected by SA in different plants. Levels of some metabolites such as proteins and lipids depend on temperature in plants. Low temperature affects the expression of some genes, which are related to protein and lipid production in plants [30]. Breaking dormancy causes distinctive changes in the pattern of peptide stains either in quality or quantity, and the number of polypeptides decreases [31]. Studying changes in nucleic acid, soluble proteins, nitrogen content, amino acids, and protease activity during transferring dormancy period to active growth including flower and leaves formation and differentiation indicates that apical bud growth and development resumption need an increase in metabolical activity [32]. Since soluble protein influence on saffron corm growth and differentiation processes in a great extent, Bagri et al. [33] indicated that changing in free amino acids pool in corm tissue occurs to break down some storage proteins metabolism which influence the dormancy break down and sprouting process of saffron corm. Bud development and vegetative to productive transition in saffron need protein components which are fully supported by corm storage [32].

### 3.3. Enzyme

Since no abiotic stress was applied on corms and CAT, APX, PPO, and GPX enzymes were measured in the same growth stage, no significant difference was detected between treatments (Table 1).

Ebrahimzadeh and Abrishamchi [34] declared that differences in catalase, peroxidase, and polyphenoloxisdse activities during the dormancy and different stage of growth were significant. However, our experiment was done in a specified stage of growth, and maybe because of that, data had showed no differences.

There was a significant difference (*P* < 0.05) in PAL enzyme activity (Table 1). Treatments were divided into two groups. Pre-cold treatment had the highest amount of PAL activity, and the other group included SA1% and control treatment with the lowest amount of PAL activity (Figure 1(c)).

PAL is the first enzyme which is involved in cinnamate-related secondary metabolism in plants. Under stressed growth conditions, an induction in the stage of flowering occurs as well as an increase in PAL activity [35]. The important and diverse role of SA as a signaling molecule in plants has been proven. It can regulate the physiological, biochemical, and molecular processes in plants and accordingly can influence on the growth, development, and production of secondary metabolites [36]. Tajik et al. [37] demonstrated that an increase in SA level leads to an increase in *PAL* gene activity in saffron leaves.

Correlation analysis showed that day to flowering trait in our experiment had a negative correlation (–0.587^*∗*^) with the amount of PAL activity (Table 2). It means that PAL activity increment can stimulate flowering as it has mentioned in the previous section. The SA2%, SA1%, and pre-cold treatment allocated the lowest period to flowering and control treatment which had the lowest amount of PAL activity making a delay in the flowering. Some compounds in PAL-regulated metabolism pathways may act as flowering stimuli. Chlorogenic acid (CGA), which is in the category of phenylpropanoids, is considered as a plant flowering stimulation which is regulated by PAL [24]. PAL enzyme is known as a biochemical marker of secondary metabolism under different types of stresses or in response to plant growth regulators such as SA [38].

### 3.4. Total Sugar and Starch

The experiment showed that the applications of SA and pre-cold treatment were effective on inducing the production of total soluble sugar and starch in corms of saffron. ANOVA results for both traits showed significant differences between treatments at 1% (Table 1). In terms of corm total sugar content, treatments were categorized in two groups, the control treatment and SA2% with the highest amount of sugar, whether SA1% and pre-cold treatment placed in another group (Figure 1(d)). The highest amount of starch content was belonged to pre-cold treatment, while the control treatment had the lowest amount of starch content (Figure 1(e)).

The flowering time is an important stage in the plant life cycle, and this time is highly affected by environmental and interior signals in plants. One of the most interior important signals is the whole plant carbon status [39]. Since sugars metabolism occurs promptly, this metabolite could swap according to the environmental condition. Rosa et al. [40] and Gibson [41] declared that sugar can act as a primary messenger and has a key role in the controlling genes expression. Heyer et al. [42] stated that sugar content in the plant apex had an extreme effect on flowering time. The relation between flowering stage and sugar metabolism and signaling in plants was also intimated by King [43]. Some researchers notified that sugar and plant hormones could interact with each other [44]. Proteomic studies showed that exogenously SA application in plants could effect on some proteins and enzymes regulation which are related to carbohydrate metabolism [45]. Our results are supported by the finding of Dong et al. [46] which demonstrated that the SA effects on sugar metabolism and starch content in cucumber and Shehata et al. [47] which indicated that SA affects sugar content conflictingly in a way that lower SA concentration (below 2.5 mM) could increase seeds total sugar content.

Applying SA in plants could enhance starch and polysaccharide contents [48]. The same as our result Kang et al. [49] reported reduction in the number of starch granules and some other ultrastructure changes in banana leaves after applying SA. The *α*-amylase activity, the effective enzyme on starch granules, increased in seeds which were treated by SA, and then it can effect on plant starch content accordingly [50]. Also, our finding was accordance with Dong and Beckles [51] that expressed that the higher starch accumulation in plants can be results of low temperature, as well as Rosa et al. [40] that reported an increment in starch content of Quinoa cotyledon when incubated at 5°C for two days.

Burešová et al. [52] pointed out the negative correlation between starch and protein content in their experiments, which is corresponded by the assessed value of correlation coefficient in the present study (Table 2).

Data analysis showed a negative correlation between sugar and starch content in saffron corms (Table 2). According to Dong and Beckles [51], the proportion between starch and sugar content in plants is changing constantly. During flowering stage, saffron corm acts as a carbohydrates source. In the other words, starch content reduces and converts to sugar in order to supply flowering process. The effect of SA and pre-cold treatments in comparison to control treatment and their significant correlation on sugar and starch content with flowering time, which is one of the important traits in saffron, was obvious in this study and led to earlier flowering.

## 4. Conclusion

The important result of this experiment was the effect of different levels SA and pre-cold treatment on flower stimuli in saffron and their effects on PAL enzyme activity and corm protein, starch, and total sugar content. Although our result shows that even low temperature can improve the day to flowering trait in comparison to control corms, other data, which were not listed here, showed that pre-cold treatment was not successful to improve quality and quantity of other traits, which are related to saffron flower.

Consequently, it is advised to use exogenous SA in appropriate doses to achieve an earlier date of flowering, and according to other researchers, it can also improve saffron yield and quality.

## Figures and Tables

**Table 1:** Variance analysis for the effect of SA and pre-cold treatment on day to flowering and enzymatic traits of saffron.

Mean square								
S.O.V	APX	CAT	PAL	PPO	Day to flowering	Protein	Sugar	Starch
Treatment	7.827 × 10-7 ns	1.2259 × 10-7 ns	1.458^*∗∗*^	0.01 ns	73.111^*∗∗*^	887.874^*∗*^	5493.208^*∗∗*^	7653.865^*∗∗*^
Error	0.00001	2.9342 × 10–7	0.264	0.004	1.167	211.378	713.649	474.679

ns not significant. ^*∗*^ and ^*∗∗*^Significant at *P* ≤ 0.01 and *P* ≤ 0.05, respectively.

**Table 2:** Correlation between APX, CAT, GPX, PAL, PPO, day to flowering, stretch, and sugar content.

Correlations									
	APX activity	CAT activity	GPX activity	PAL activity	PPO activity	Day to flowering	Protein	Starch	Sugar
APX activity		−0.12^ns^	−0.117^ns^	0.05^ns^	−0.234^ns^	−0.338^ns^	−0.045^ns^	0.278^ns^	−0.348^ns^
CAT activity			−0.438^ns^	−0.197^ns^	−0.149^ns^	−0.056^ns^	0.225^ns^	−0.159^ns^	−0.147^ns^
GPX activity				0.257^ns^	0.672^*∗*^	0.084^ns^	−0.634^*∗*^	0.424^ns^	−0.239^ns^
PAL activity					0.437^ns^	−0.587^*∗*^	−0.349^ns^	0.678^*∗*^	−0.343^ns^
PPO activity						−0.128^ns^	−0.893^*∗∗*^	0.607^*∗*^	−0.402^ns^
Day to flowering							0.229^ns^	−0.608^*∗*^	0.522^ns^
Protein								−0.688^*∗*^	0.616^*∗*^
Starch									−0.677^*∗*^

ns not significant. ^*∗*^Correlation is significant at the 0.05 level. ^*∗∗*^Correlation is significant at the 0.01 level.

## Data Availability

Data are available upon request.
